# Transcriptome profiling reveals an integrated mRNA–lncRNA signature with predictive value for long-term survival in diffuse large B-cell lymphoma

**DOI:** 10.18632/aging.104100

**Published:** 2020-11-18

**Authors:** Qian Gao, Zhiyao Li, Lingxian Meng, Jinsha Ma, Yanfeng Xi, Tong Wang

**Affiliations:** 1 Department of Health Statistics, School of Public Health, Shanxi Medical University, Taiyuan 030001, China; 2 Department of Pathology, Shanxi Cancer Hospital, Taiyuan 030013, China

**Keywords:** diffuse large B-cell lymphoma, long-term survival, mRNA-lncRNA signature, predictive accuracy

## Abstract

For patients with diffuse large B-cell lymphoma (DLBCL), survival at 24 months is a milestone for long-term survival. The purpose of this study was to develop a multigene risk score (MGRS) to refine the International Prognostic Index (IPI) model to identify patients with DLBCL at high risk of death within 24 months. Using a robust statistical strategy, we built a MGRS incorporating nine mRNAs and two lncRNAs. Stratification and multivariable Cox regression analysis confirmed the MGRS as an independent risk factor. A nomogram based on IPI+MGRS model was constructed and its calibration plot showed close agreement between predicted 2-year survival rate and observed rate. The 2-year AUC was bigger with the IPI+MGRS model (ΔAUC=0.162; 95%CI 0.1295–0.1903) than with the IPI model, and the IPI+MGRS model more accurately predicted the prognostic risk of DLBCL. The 2-year survival decision curve revealed the IPI+MGRS model was more useful clinically than the IPI model. Functional enrichment analysis showed that the MGRS correlated with cell cycle, DNA replication and repair. The results were validated using an independent external dataset. In conclusion, we successfully developed an integrated mRNA–lncRNA signature to refine the IPI model for predicting long-term survival of patients with DLBCL.

## INTRODUCTION

Diffuse large B-cell lymphoma (DLBCL) is a major subtype of non-Hodgkin’s lymphoma characterized by remarkable clinical and biological heterogeneity [1]. Although most patients are cured with upfront chemoimmunotherapy, approximately 40% of patients have an adverse prognosis [2]. If high-risk patients can be identified prospectively, they could receive more effective therapy. Recent studies have highlighted that the occurrence of adverse events (relapse, progression, death, etc.) within 24 months from diagnosis is decisive for the survival outcome of patients with DLBCL [3–6]. Maturer et al. [6] and Jakobsen et al. [5] showed that patients who achieved event-free survival at 24 months and Maturer et al. [4] showed that patients who achieved progression-free survival at 24 months had similar overall survival to DLBCL-free individuals in the general population. Ekberg et al. [3] found that the remaining life expectancy of patients who survived the first 24 months after diagnosis was close to that of the general population. Together, these findings implied that most of the high-risk patients with DLBCL experienced an adverse event in the first 24 months after diagnosis. Therefore, accurate prediction of early events is critical in detecting high-risk patients with DLBCL.

Clinical prognostic scores, including the International Prognostic Index (IPI), age-adjusted IPI, revised IPI, and National Comprehensive Cancer Network IPI [7], have been used to estimate the survival chance of patients with DLBCL beyond a certain time point. In 2018, treatment decisions still relied mainly on clinical factors outlined in the IPI [8]. However, these clinical prognostic models fail to reliably predict the clinical course of lymphoma and patients with identical prognostic scores often have variable outcomes [2]. This finding highlights the need for more precise, patient-specific, and biologically-based biomarkers to predict outcomes of DLBCL. Specific genetic alterations and abnormal protein abundance were found to partially explain the diverse outcome of DLBCL [9, 10]. For example, DLBCLs with MYC rearrangements and BCL2 and/or BCL6 translocations (double/triple hit) have a poor prognosis [9, 11, 12]. The combination of MYC rearrangements and inactive TP53 mutations adversely affected the patients’ overall survival [8, 12, 13]. DLBCLs with overabundance of both the MYC and BCL2 proteins also have been associated with poor prognosis [8, 12]. Abnormal gene expression is considered as another factor that can be used independently of the IPI to predict the outcome of DLBCLs [14–17]. Several mRNA-based molecular signatures have been developed for DLBCL prognostic stratification [15, 17, 18]. However, Hong et al. [19] evaluated the performance of some of these signatures and found that they provide limited added value in risk assessment of DLBCLs. These findings implied that other undiscovered RNA signatures may help to explain the heterogeneity of outcomes in patients with DLBCL.

Long non-coding RNAs (lncRNAs) are >200-nt long RNAs that are involved in multiple biological processes, including cell differentiation and development [20–22]. Mutations and misregulation of lncRNAs have been found to promote tumorigenesis and metastasis in various types of cancer [23], and thousands of lncRNAs have been reported to be abnormally expressed in DLBCLs compared with their expression in normal B-cells [24]. Cheng et al. [25] demonstrated that upregulation of lncRNA *TUG1* had an oncogenic role in DLBCL by inhibiting the ubiquitination of MET. LncRNA *MALAT1* was found to promote tumorigenesis and immune escape of DLBCLs by sponging the microRNA miR-195 [26], and high expression levels of lncRNA *NEAT1_1* were shown to be associated with poor prognosis of DLBCL [27]. These findings indicated that lncRNAs may be promising novel biomarkers for DLBCL diagnosis and prognosis.

Gene signatures that integrate mRNAs and lncRNAs have been suggested to have good prognostic value in breast and colon cancers [28, 29]. We considered that combinations of mRNAs and lncRNAs may improve risk prediction for patients with DLBCL. Therefore, the purpose of this study was to develop and validate an integrated mRNA–lncRNA signature that could refine the IPI model for early event/long-term survival prediction.

## RESULTS

### Characteristics of the datasets used in this study

A total of 1244 patients from five Gene Expression Omnibus (GEO) datasets (https://www.ncbi.nlm.nih.gov/geo/) were selected and comprehensively studied. The characteristics of the training dataset and the validation dataset and its components are summarized in Supplementary Table 1. We used GSE10846, which included 412 patients with DLBCL, as the training dataset; 163 of them had died (event) and 122 of them (122/163) died within the first 2 years after diagnosis. The validation dataset (termed ComBatData) was an integrated dataset that included 832 patients with DLBCL; 470, 69, 221, and 72 were from GSE31312, GSE23501, GSE87371, and GSE98588, respectively. Among them, 263 patients had died (event) and 184 of them died within the first 2 years after diagnosis. The four datasets were merged using the ComBat method.

### Identification of RNAs associated with long-term survival

**Figure 1:**
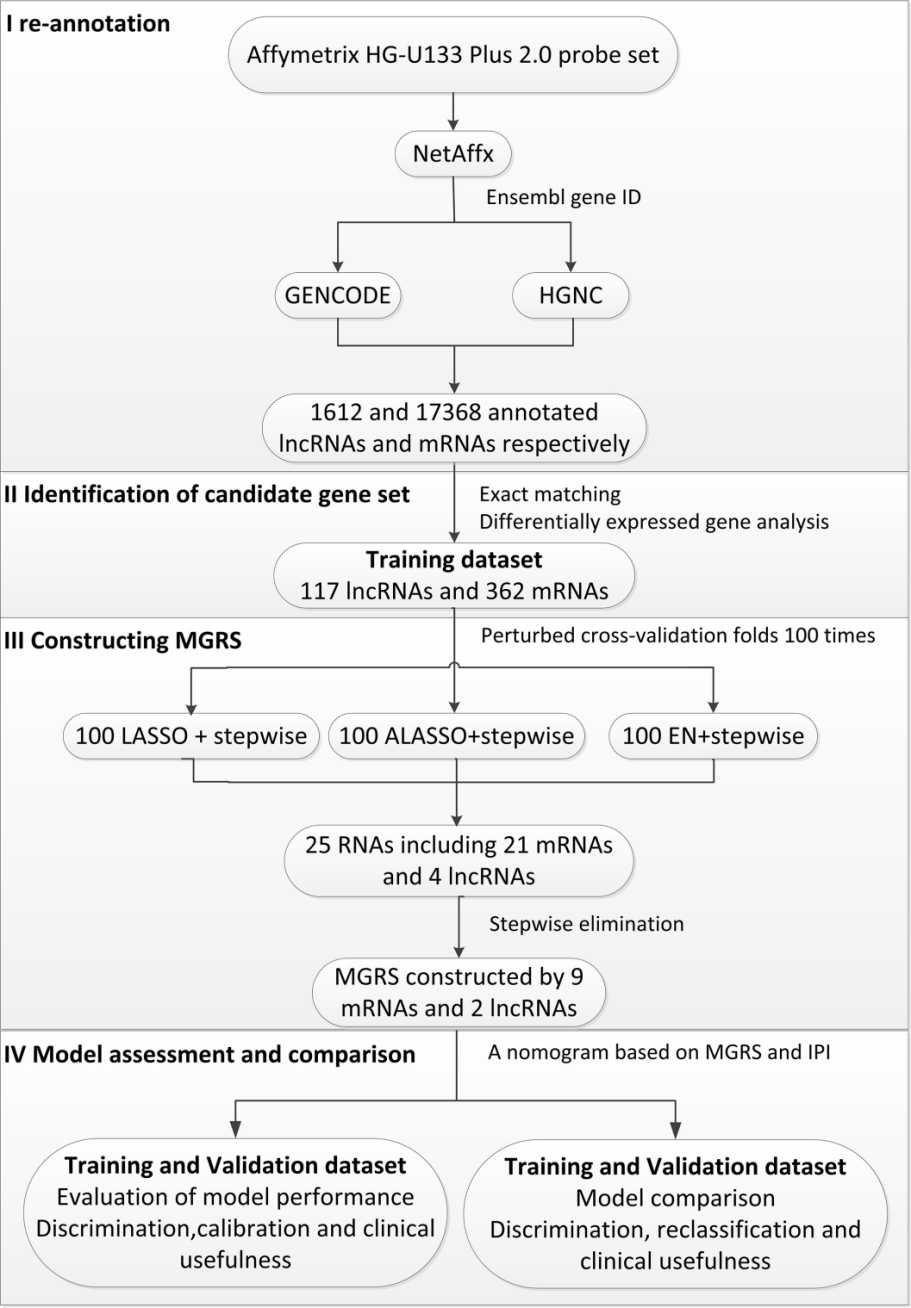
**Flow chart of the statistical process used in this study.**

**Figure 2:**
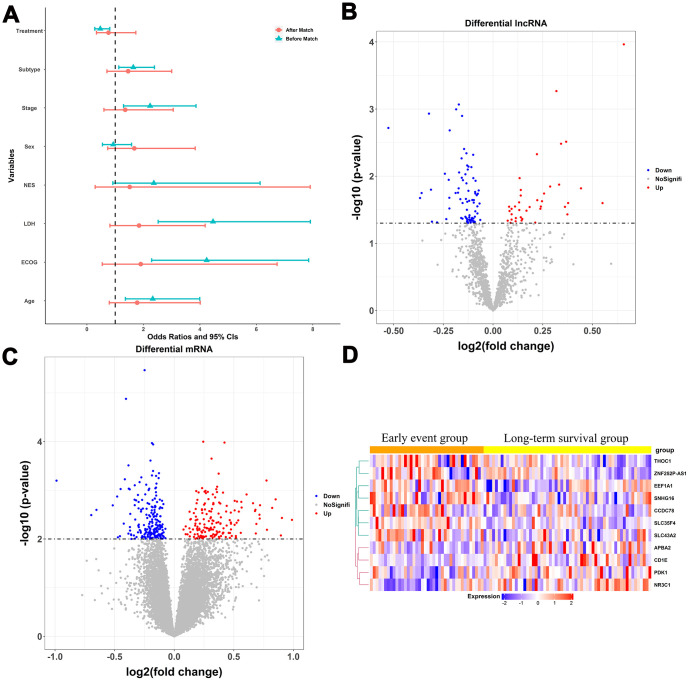
**Construction of the multigene risk score (MGRS).** (**A**) Baseline characteristics of patients in the early event and long-term survival groups before and after matching; ECOG, Eastern Cooperative Oncology Group; LDH, lactate dehydrogenase; NES, number of extra-nodal sites; Stage, Ann Arbor stage; (**B**, **C**) Volcano plots for differentially expressed lncRNAs and mRNAs in the long-term survival group compared with the early event group; (**D**) Expression patterns of the 11 RNAs included in the MGRS.

The statistical process used in this study is illustrated in Figure 1. We divided the patients in the training dataset into an early event group and long-term survival group according to their survival time and status (death). To minimize confounding by baseline characteristics and to derive credible differentially expressed genes, we balanced baseline features between the two groups by exact matching analysis (Figure 2A). Before matching, age, Eastern Cooperative Oncology Group, Ann Arbor stage, treatment (CHOP vs. RCHOP), subtype (germinal center B-cell-like, GCB vs. non-GCB), and lactate dehydrogenase concentration were significantly different between the two groups. After matching, the baseline characteristics were well-balanced (Figure 2A). The volcano plot (Figure 2B, 2C) shows that 479 RNAs comprising 117 lncRNAs and 362 mRNAs were differentially expressed between the two groups; among them, 38 lncRNAs and 163 mRNAs were upregulated in the long-term survival group compared with the early event group. To construct the gene risk model, we used the 100 times procedure of the penalized Cox regression+stepwise and screened 25 RNAs (Table 1 and Figure 1; see Section 4.4 for details of the screening process). Stepwise elimination reduced the 25 RNAs to a subset of 11 RNAs (nine mRNAs and two lncRNAs), which was used in the final gene risk model. As shown in Table 1, nine of the 11 RNAs were selected 100 times in at least one penalized regression method; the other two RNAs were selected less frequently but exceeded 60 times in one penalized regression method. This result implied that the genes included in the final model were relatively insensitive to the regularization level of the penalized regression. The expression patterns of the 11 genes are presented in Figure 2D. The expression levels of five of the nine mRNAs, *THOC1*, *EEF1A1*, *CCDC78*, *SLC35F*4, and *SLC43A2*, and the two lncRNAs, *ZNF252P-AS1* and *SNHG16*, were relatively low in the long-term survival group, whereas the expression levels of four of the mRNAs, *CD1E*, *APBA2*, *PDK1*, and *NR3C1*, were relatively high in the long-term survival group.

**Table 1:** Genes screened using the penalized regression method.

**Gene name**	**gene type**	**Selected times**	
		**LASSO**	**ALASSO^†^**	**EN^‡^**
**ALDOC**	mRNA	100	45	100
**ANOS1**	mRNA	100	100	100
**APBA2**	mRNA	100	100	100
**CCDC78**	mRNA	100	45	100
**CD1E**	mRNA	100	100	100
**DMD**	mRNA	100	37	100
**JAML**	mRNA	100	37	100
**NRROS**	mRNA	100	45	100
**SLC22A1**	mRNA	100	37	100
**SLC35F4**	mRNA	100	100	100
**SLC43A2**	mRNA	100	100	100
**SNHG16**	lncRNA	100	100	100
**THOC1**	mRNA	100	100	100
**ZNF252P-AS1**	lncRNA	100	100	100
**POMZP3**	mRNA	72	0	100
**TSPOAP1-AS1**	lncRNA	72	63	46
**ONECUT1**	mRNA	71	37	6
**CBFA2T3**	mRNA	57	37	94
**EEF1A1**	mRNA	57	100	94
**DDX11-AS1**	lncRNA	43	0	6
**GSTA4**	mRNA	29	0	94
**NR3C1**	mRNA	0	63	0
**PDK1**	mRNA	0	63	0
**FAM49B**	mRNA	0	0	54
**DCAF5**	mRNA	0	0	2

^†^ALASSO: adaptive LASSO

^‡^EN: elastic net

### Multigene risk score

The multigene risk score (MGRS) was defined as the prognostic index of multivariable Cox models constructed with the 11 selected RNAs. The MGRS can be represented as follows:

MGRS = −0.445 × *CD1E* + 0.243 × *ZNF252P-AS1* − 0.346 × *APBA2* + 0.258 × *THOC1* + 0.346 × *SNHG16* − 0.312 × *NR3C1* + 0.213 × *SLC35F4* − 0.318 × *PDK1* + 0.202 × *CCDC78* + 0.245 × *SLC43A2* + 0.227 × *EEF1A1*.

**Figure 3:**
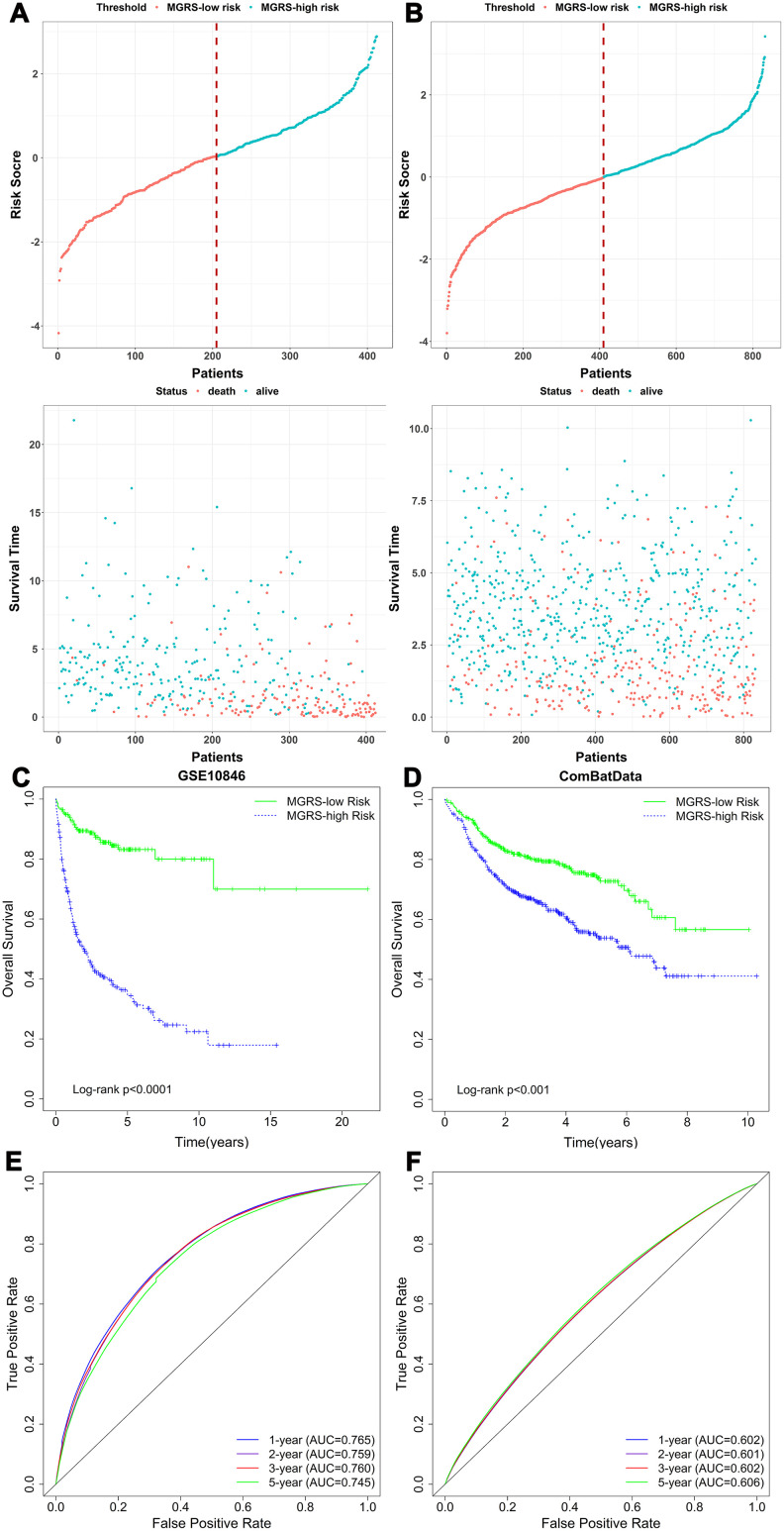
**The relationship between multigene risk score (MGRS) and overall survival of patients with DLBCL.** Distribution of MGRS and survival status in (**A**) the training dataset and (**B**) the validation dataset; (**C**, **D**) Kaplan–Meier survival curves of MGRS-high risk and MGRS-low risk groups in the training and validation datasets; (**E**, **F**) Time-dependent ROC curves at 1, 2, 3, and 5 years after diagnosis for the MGRS in the training and validation datasets.

The MGRS was calculated for each patient in the training dataset. The mean of the MGRSs was 0, which was defined as the cutoff value for dividing patients into MGRS-high risk or MGRS-low risk groups. Figure 3A shows the distribution of the MGRSs and survival status in the training dataset. The results indicate that patients with higher MGRSs had worse overall survival than patients with lower MGRSs. The 2-year survival rate for the patients with the higher MGRSs was 49.8% compared with 89.0% for patients with the lower MGRSs (hazard ratio (HR)=5.975, 95% CI 3.995–8.938, p <0.001; Figure 3C). The time-dependent receiver operating characteristic (ROC) curves at 1, 2, 3, and 5 years after diagnosis are shown in Figure 3E. The area under the ROC curve (AUC) at 2 years was 0.759.

The same analyses were conducted for the validation dataset (Figure 3B, 3D, 3F). Using the cutoff value determined with the training dataset, 410 (49.3%) and 422 (50.7%) patients were assigned to the MGRS-low and MGRS-high risk groups, respectively. The 2-year survival rates were 70.9% for patients in the MGRS-high risk group and 83.3% for patients in the MGRS-low risk group (HR=1.882, 95% CI 1.463–2.422, p <0.001; Figure 3D). The 2-year AUC for the validation dataset was 0.601.

Together, these results suggest that the MGRS has potential value in predicting 2-year survival of patients with DLBCL.

### Independence of the MGRS in predicting long-term survival

**Figure 4:**
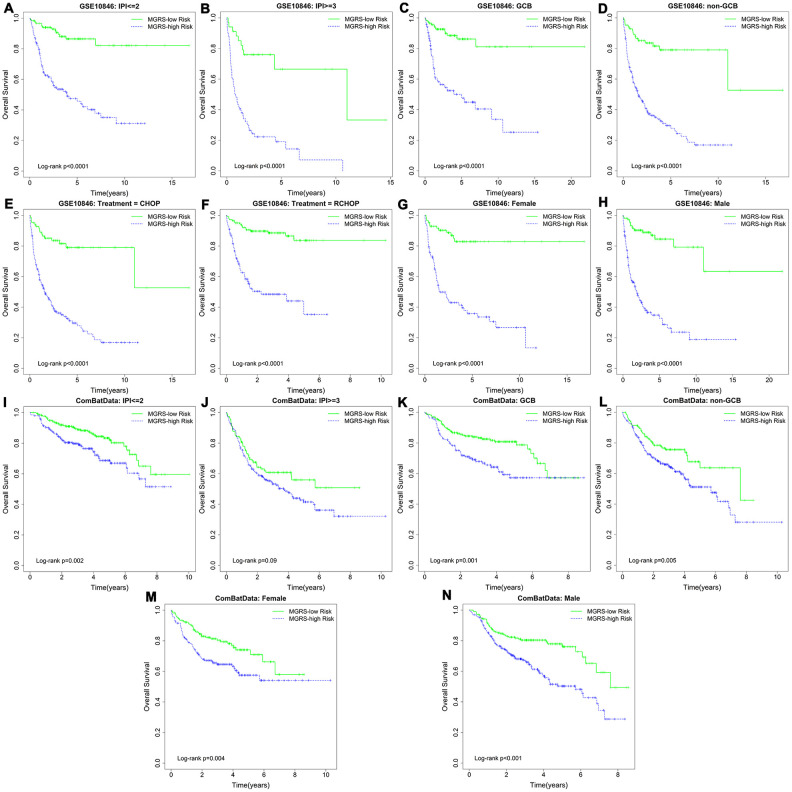
Kaplan–Meier survival curves of multigene risk score (MGRS)-high risk and MGRS-low risk groups stratified by clinical factors in the (**A**–**H**) training dataset and (**I**–**N**) validation dataset.

Stratification analysis and multivariable Cox regression analysis were conducted to explore the independent role of the MGRS in predicting long-term survival. Patients in the training dataset were stratified based on the IPI (IPI ≥3/IPI ≤2), sex (female/male), subtype (GCB/non-GCB), and treatment (CHOP/RCHOP). Figure 4A–4H shows the Kaplan-Meier survival curves for the MGRS-high risk and MGRS-low risk groups within each stratum, which demonstrated that, in each subgroup, patients in the MGRS-high risk group had significantly worse prognosis than patients in the MGRS-low risk. Similar results were obtained for patients in the validation dataset (Figure 4I–4N). There were significant differences in overall survival between patients in the MGRS-high risk and MGRS-low risk groups in each subgroup, except for patients in the IPI ≥3 subgroup (p=0.09). However, the survival curve for the MGRS-high risk group was lower than the survival curve for the MGRS-low risk group in the IPI ≥3 subgroup, and the median survival time for patients in the MGRS-high risk group was 3.69 years. The median survival time for patients in the MGRS-low risk group was not reached.

The hazard ratios (HRs) and corresponding model coefficients for the univariate and multivariable Cox model with stepwise procedure are summarized in Table 2. For the multivariable Cox regression model, we found that, after adjusting for the MGRSs, the HR of the IPI score was moderately reduced, suggesting that the 11 RNAs contained prognostic information that was at least partially independent of the IPI.

**Table 2:** Univariate and multivariable Cox regression with the training and validation datasets.

**Variables**	**Univariate analysis**					**Multivariable analysis**				
	**β**	**SE (β)**	**HR (95%CI)**	**Wald χ^2^**	**P**	**β**	**SE (β)**	**HR (95%CI)**	**Wald χ^2^**	**P**
**GSE10866(n=412)**										
MGRS	1.000	0.080	2.718 (2.319-3.185)	152.60	4.68E-35	0.940	0.090	2.560 (2.145-3.057)	108.07	2.60E-25
IPI (0-2 *vs.* 3-5)	1.067	0.178	2.907 (2.051-4.120)	35.97	2.00E-9	0.877	0.181	2.403 (1.686-3.423)	23.56	1.21E-6
Subtype (GCB vs. non-GCB)	0.854	0.172	2.349 (1.676-3.293)	24.54	7.28E-7					
Treatment (RCHOP *vs.* CHOP)	-0.657	0.167	0.518 (0.374-0.719)	15.48	8.33E-5					
Sex (female *vs.* male)	0.010	0.163	1.010 (0.734-1.389)	0	0.951	-	-	-	-	-
**CombatData (n=832)**										
MGRS	0.340	0.057	1.405 (1.256, 1.571)	35.39	2.70E-09	0.233	0.061	1.262 (1.121, 1.422)	14.72	0.0001
IPI (0-2 *vs.* 3-5)	1.094	0.131	2.985 (2.310, 3.859)	69.78	6.63E-17	0.963	0.135	2.620 (2.010, 3.415)	50.81	1.02E-12
Subtype (GCB *vs.* non-GCB)	0.455	0.133	1.576 (1.214, 2.045)	11.66	0.0006	-	-	-	-	-
Sex (female *vs.* male)	0.067	0.133	1.069 (0.825, 1.387)	0.26	0.613	-	-	-	-	-

**Notation:** HR, hazard ratio; SE, standard error; CI, confidence interval; IPI, International Prognostic Index; GCB, germinal center B-cell-like; CHOP: cyclophosphamide, doxorubicin hydrochloride, vincristine, and prednisone; RCHOP: rituximab, cyclophosphamide, doxorubicin hydrochloride, vincristine, and prednisone.

### Evaluation and comparison of model performances

**Figure 5:**
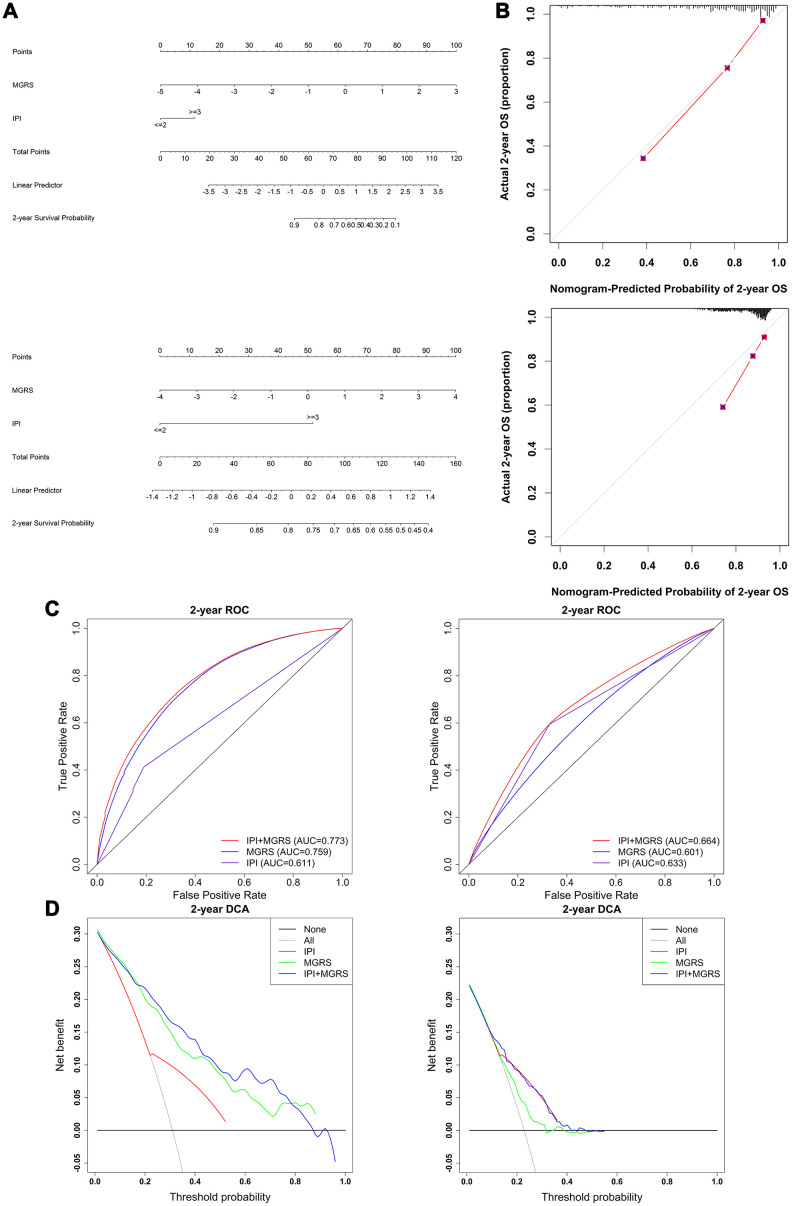
**Evaluation and comparison of model performances in predicting 2-year survival.** (**A**) Nomogram based on the International Prognostic Index (IPI) and multigene risk score (MGRS) with the training dataset (top panel) and validation dataset (bottom panel); (**B**) Calibration plot of the nomogram for estimation of survival rates at 2 years after diagnosis in the training dataset (top panel) and validation dataset (bottom panel); (**C**) Time-dependent ROC curves at 2 years after diagnosis for the IPI, MGRS, and IPI+MGRS models in the training dataset (left panel) and validation dataset (right panel); (**D**) Decision curves at 2 years after diagnosis for the IPI, MGRS, and IPI+MGRS models, in the training dataset (left panel) and validation dataset (right panel).

Nomograms were constructed for the training and validation dataset based on the results of the multivariable Cox regression model (Figure 5A). A nomogram is a quantitative tool that can be used in a clinical setting to predict 2-year survival rates of patients with DLBCL. Calibration plots of the nomogram with the training and validation datasets is shown in Figure 5B. The calibration plots showed that IPI+MGRS performed well with only a slight overestimation of the 2-year survival rate for the validation cohort in one of the three groups that were obtained by dividing the samples according to the quantile of the predicted absolute risk. Figure 5C shows the 2-year ROC curves for the IPI, MGRS, and IPI+MGRS models. With the training dataset, the AUC value at 2 years after diagnosis increased from 0.611 for the IPI model to 0.773 for the IPI+MGRS model (ΔAUC=0.162, 95% CI 0.1295–0.1903). The results were similar to the validation dataset; the AUC value at 2 years after diagnosis increased by 0.031 (95% CI 0.025–0.036) for the IPI+MGRS model compared with the value for the IPI model. A decision curve was used to assess the clinical usefulness of the nomogram. As shown in Figure 5D, the IPI+MGRS and IPI models both derived more net-benefit than the other two schemes: none of the patients were at 2-year death risk or all of the patients were at 2-year death risk. The net benefit of the IPI+MGRS model was higher than that of the IPI model.

To further investigate the added predictive value from the MGRS, the category-free net reclassification index (NRI >0) was calculated to assess how much better the IPI+MGRS model was at predicting the 2-year death risk compared with the IPI model. For the training dataset, the IPI+MGRS model had an NRI (>0) of 0.894 (95% CI 0.6760–1.1211) and, for the validation dataset, the IPI+MGRS model had an NRI (>0) of 0.329 (95% CI 0.1675–0.4934).

The generalizability of the MGRS was investigated by sensitivity analysis using three different subsets of the validation dataset (termed ComBatData). The selected GEO dataset with the largest sample size (GSE31312) was defined as ComBatData1 (n=470); ComBatData without the GSE31312 dataset was defined as ComBatData2 (n=362); and ComBatData without datasets with sample sizes less than 100 (GSE98588 and GSE23501) was defined as ComBatData3 (n=691). The increments of AUC obtained by the MGRS were 0.038 (95% CI 0.0289–0.0468) for ComBatData1, 0.030 (95% CI 0.0224–0.0364) for ComBatData2, and 0.035 (95% CI 0.0284–0.0414) for ComBatData3. The NRIs (>0) for the IPI+MGRS model were 0.312 (95% CI 0.0994–0.5340), 0.325 (95% CI 0.0623–0.5780), and 0.337 (95% CI 0.1587–0.5101) for ComBatData1, 2, and 3, respectively.

These findings suggested that the model that combined IPI with MGRS had better predictive accuracy than the model that used only the IPI.

### Functional role of the MGRS

**Figure 6:**
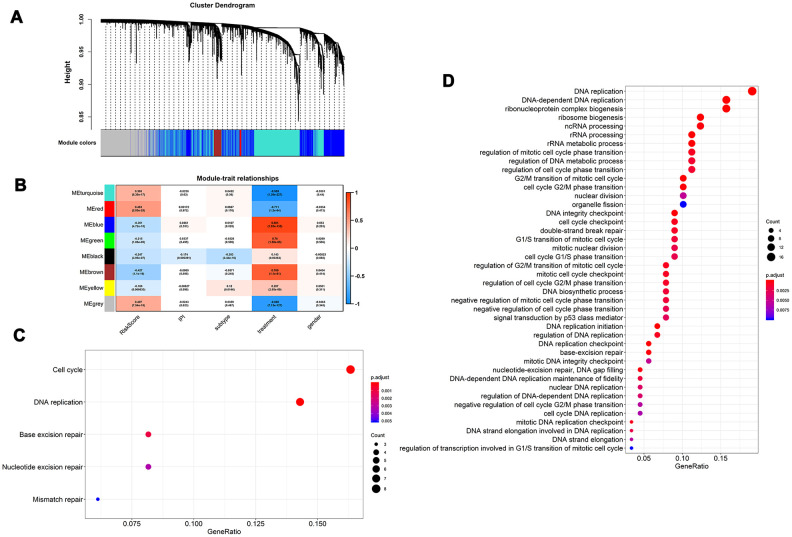
**Functional role of multigene risk score (MGRS).** Weighted gene co-expression network analysis: (**A**) Clustering dendrogram of genes and modules; (**B**) Correlation between gene modules and MGRS, clinical factors. The module marked in red had the highest correlation with MGRS; (**C**, **D**) Gene ontology (GO) and KEGG pathway analysis of the genes in the highly-correlated module.

To better understand the biological mechanism of the MGRS, we performed a weighted correlation network analysis to develop a co-expression network based on gene expression profiling. The co-expression network had eight modules (Figure 6A), and the module marked in red (Figure 6B) had the highest correlation with MGRS. The genes in this module were functionally annotated with Gene Ontology (GO) terms under the biological process category and KEGG pathways. The results indicated that the MGRSs were strong associated with cell cycle, DNA replication, and DNA repair (Figure 6C, 6D).

## DISCUSSION

Several previous studies have identified molecular markers based on gene expression profiles for improving the predictive abilities of the IPI. For example, in 2004, Lossos et al. [15] constructed a predictive model based on the expression of six genes, the Lymphoma/Leukemia Molecular Profiling Project reported a three-component signature (about 400 genes) as a risk predictor [18] and, in 2011, Alizadeh et al. [17] further simplify the prognostic model to two genes. Hong et al. [19] assessed the usefulness of these gene-expression based signatures using discrimination and reclassification metrics and found that the improvement obtained by adding a gene expression-based signature to the IPI model was limited. Considering the important role of lncRNAs in the development and prognosis of cancers [23–27], in 2016, Sun et al. [30] built a prognostic model based on six lncRNAs. However, they assessed the predictive significance based only on the p values of a multivariable Cox regression. This type of assessment is not sufficient to evaluate the prediction accuracy of a prognostic model and to quantify the incremental usefulness offered by their six-lncRNA signature. The prediction capability of such a model should be assessed internally and externally in terms of discrimination and calibration [31–33]. To assess the added usefulness offered by new markers, improvement of discriminative ability and net reclassification metric also need to be calculated to determine how much better a model with new markers is at predicting risk compared with the model without new markers [32–34]. Importantly, no previous study combined mRNAs and lncRNAs to construct a signature to predict early event and long-term survival of patients with DLBCL. Considering these limitations, we reanalyzed the transcriptome data of DLBCL and evaluated the added value of integrating a mRNA–lncRNA signature using discrimination, clinical usefulness, and reclassification metrics.

Our robust statistical strategy produced a MGRS based on nine mRNAs and two lncRNAs. Stratification analysis and multivariable Cox regression analysis revealed that the MGRS provided prognostic information that was independent of the IPI. According to the results of multivariable Cox analysis, a nomogram was constructed by integrating the IPI and MGRS. The prediction efficiency of the nomogram was confirmed by the calibration plot. The nomogram may help clinicians identify high-risk patients with DLBCL. To evaluate the added value from the MGRS in 2-year survival prediction, we compared the performances of the IPI+MGRS and IPI models by assessing their discrimination, reclassification, and clinical usefulness. The addition of the MGRS to the IPI improved the discrimination and net reclassification performance. The decision curve showed that the IPI+MGRS model had better clinical practicality than the IPI model. Together, these results indicated that the MGRS can be used to refine the IPI model. These results were validated with an independent external dataset. However, the added value offered by the MGRS for the validation dataset was modest compared with the added value for the training dataset. This may be because the development of the MGRS relied on the training dataset [35] and the sample mix in the validation dataset was different but related to the training samples [36]. Sensitivity analysis showed similar results to the validation dataset. These findings indicated the broad applicability of the MGRS in DLBCL.

The functional enrichment analysis revealed a strong association between the MGRS and cell cycle, DNA replication, and DNA repair. We investigated the relationship between the genes included in the MGRS and cancer (summarized in Table 3). Six of the nine mRNAs included in the signature have been reported to be associated with cancer. *PDK1* encodes pyruvate dehydrogenase kinase 1, which inactivates the pyruvate dehydrogenase (PDH) enzyme complex that converts pyruvate to acetyl-coenzyme A, thereby inhibiting pyruvate metabolism via the tricarboxylic acid (TCA) cycle [37]. Thomas et al. [38] showed that inhibition of the PDH enzyme complex caused by increased *PDK1* expression was associated with the Warburg metabolic and malignant phenotype of cancer, and that knockdown of *PDK1* decreased invasiveness and inhibited tumor growth. The prognostic performance of *PDK1* varied among different cancers. Overexpression of *PDK1* has been associated with poor prognosis in non-small cell lung cancer [39], nasopharyngeal carcinoma [40], and head and neck squamous cancer [41], whereas Shinkyo et al. [42] found that increased expression of *PDK1* prolonged survival in colon cancer, which is consistent with our findings. The inconsistency in prognostic values for different cancers is poorly understood and needs further investigation. Eukaryotic translation elongation factor 1A1 (eEF1A1), encoded by *EEF1A1*, is an evolutionarily conserved elongation factor protein that triggers the initiation of protein translation elongation [43]. eEF1A1 is involved in multiple biological processes, including cytoskeletal remodeling, proteasome-mediated protein degradation, and control of cell cycle, growth, and death [44]. Aberrantly upregulated eEF1A1 has been detected in many tumor tissues and overexpression of eEF1A1 is related to cancer cell proliferation, invasion, and migration [45]. *NR3C1* encodes glucocorticoid receptor (GR), which was shown to be involved in inflammatory responses, cellular proliferation, and differentiation in target tissues [46]. The expression of *NR3C1* was found to be reduced in many tumor tissues owing to methylation of its promoter [47, 48]. For prognostic performance, high GR expression levels were associated with poor outcomes in estrogen-negative (ER−) breast and ovarian cancers, and with prolonged survival in ER+ breast cancer [49, 50]. In this study, we identified overexpression of *NR3C1* as a predictor to predict better outcomes. *NR3C1* may be a good prognostic factor because overexpressed GR has been shown to reduce glucocorticosteroid resistance in chemotherapy [51]. THO complex 1 (Thoc1) is a nuclear matrix protein that plays important roles in transcription elongation and mRNA export [52], and increased expression of Thoc1 was found in a number of tumors and correlated with poor prognosis [53–55]. *APBA2* encodes a tumor suppressor and was found to be hypermethylated in various cancers [56–58], and *SLC43A2* was reported to be associated with gastric cancer [59]; however, the prognostic value of these two genes is unclear. We found that high expression of *APBA2* and low expression of *SLC43A2* were associated with long-term survival in patients with DLBCL. The carcinogenic and prognostic mechanisms of *APBA2* and *SLC43A2* require further exploration. Of the two lncRNA included in the prognostic signatures, only *SNHG16* has been reported to be associated with tumorigenesis and prognosis. *SNHG16* was highly expressed in several cancers and silencing it inhibited cell proliferation, migration, and invasion, and induced apoptosis [60, 61]. In agreement with our results, previous survival analysis showed that patients with high *SNHG16* expression had shorter survival times for various cancers [60, 61]. The relationships between the other three mRNAs and lncRNA *ZNF252P-AS1* and cancer have not been reported until now, so further research is needed to clarify their potential functions in cancer.

**Table 3:** Relationship between the genes included in the multigene risk score (MGRS) and cancers.

**Gene names**	**Gene type**	**Potential roles in tumorigenesis and tumor progression**	**Relationship between overexpression/ overabundance with cancer prognosis**
***PDK1***	mRNA	1. Upregulated *PDK1* was associated with Warburg metabolic and malignant phenotype of cancer [38];	1.shorter survival: non-small cell lung cancer [39], nasopharyngeal carcinoma[40], and head and neck squamous cancer [41]
		2. Knockdown of *PDK1* decreased invasiveness and inhibited tumor growth [38]	2. prolonged survival: Colon Cancer [42]
***EEF1A1***	mRNA	Overexpression of eEF1A1: related to cancer cell proliferation, invasion, and migration [45]	
***NR3C1***	mRNA	Downregulation due to methylation: breast cancer [47], colorectal tumors [48]	1. shorter survival: Estrogen-negative (ER-) breast cancer [49], ovarian cancer [50]
			2. prolonged survival: ER+ breast cancer [49]
***THOC1***	mRNA	1. Upregulated in colorectal cancer [53], breast cancer [54], and cancer cell [54]	1. shorter survival: colorectal cancer [53]
		2. High Thoc1 expression associated with prostate cancer aggressiveness and recurrence [55]	
***APBA2***	mRNA	1. Hypermethylated in gastric cancer [56], colorectal carcinoma and gastric carcinoma [57, 58]	
		2. Upregulated in early Endometrial endometrioid carcinoma [57]	
***SLC43A2***	mRNA	Associated with gastric cancer [59]	
***SNHG16***	lncRNA	1. Overexpression in non-small cell lung cancer [60] and oral squamous cell carcinoma [61].	1. shorter survival: non-small cell lung cancer [60]
		2. Associated with cancer cell proliferation, migration and invasion [60, 61]	

Our study has some limitations. First, all of the results derived in this study were based on publicly available datasets and lacked validation in a prospective clinical trial. Second, the carcinogenic and prognostic roles of the RNAs in the signature need to be validated in future studies.

In conclusion, we developed an integrated mRNA–lncRNA signature for predicting the long-term survival of patients with DLBCL. The addition of the MGRS improved the prognostic abilities of the IPI model. Future prospective clinical trials and basic research are needed to consolidate the validity of the proposed integrated mRNA–lncRNA signature.

## MATERIALS AND METHODS

### Selection of DLBCL datasets

We systematically searched the GEO database (https://www.ncbi.nlm.nih.gov/geo/) for DLBCL expression profiling studies (June 2020) with the search term “((Expression profiling by array [DataSet Type]) AND DLBCL) AND *Homo sapiens* [Organism]”. Studies were included if they met the following criteria: (i) patients were newly diagnosed with primary DLBCL; (ii) gene expression profiling were conducted in pretreatment tumor tissue using the Affymetrix HU133 Plus 2.0 microarray (HG-U133 Plus_2.0); and (iii) the IPI and overall survival information were available. Five datasets were selected, GSE10846 [18], GSE31312 [62], GSE23501 [63], GSE87371 [64], and GSE98588 [65]. The selection process is illustrated in Supplementary Figure 1. After removing patients with missing overall survival information, a total of 1244 patients with DLBCL were selected and reanalyzed. They included 412 patients from GSE10846, 470 from GSE31312, 69 from GSE23501, 221 from GSE87371 and 72 from GSE98588. GSE10846 was used as the training dataset. GSE31312, GSE23501, GSE87371 and GSE98588 were merged using the ComBat method [66] and used as the external validation dataset (termed ComBatData). ComBat is a widely used and effective method to remove potential batch effects across different studies.

### Preprocessing and re-annotating the gene expression profiles

Raw CEL files of the five selected GEO studies were downloaded from the GEO database. Each dataset was background-adjusted, normalized, and summarized using the robust multi-array average (RMA) algorithm [67]. To obtain the lncRNA and mRNA expression profiles, we re-annotated the microarray probes as described previously [68, 69]. Briefly, the Affymetrix HG-U133 Plus 2.0 probe set ID was mapped to the NetAffx Annotation Files (HG-U133 Plus 2.0 Annotations, CSV format, release 36, 07/12/16). Probe sets with an Ensembl gene ID in the NetAffx annotation were extracted. Using the Ensembl gene ID, we obtained the relationship between the probe set ID and the corresponding gene type and gene symbol using GENCODE (release 23; https://www.gencodegenes.org/) and HGNC (https://www.genenames.org/). Finally, we obtained 1612 annotated lncRNAs and 17,368 annotated mRNAs. When multiple probes were annotated to a common gene, the mean of the multiple probes was used to estimate the expression of the RNA.

### Identification of early event associated mRNAs and lncRNAs

In this study, the early event was defined as death in the first 2 years after diagnosis [3]. Patients in the training dataset were divided into an early event group and long-term survival group. The long-term survival group included patients who survived for more than 2 years and survived during follow-up. To balance the clinical characteristics between these two groups and enable a robust and credible comparison of gene expression levels, we performed exact matching analysis [70]. The variables that were matched were age, sex, Eastern Cooperative Oncology Group performance status, number of extra-nodal sites, Ann Arbor stage, lactate dehydrogenase concentration, treatment (CHOP vs. R-CHOP), and subtype (germinal center B-cell-like, GCB vs. non-GCB). Forty patients in the early event group were matched to 59 patients in the long-term survival group. Liner models and empirical Bayes methods were used to identify differentially expressed lncRNAs and mRNAs, and the thresholds were p <0.05 and p <0.01 respectively [29, 71]. The differentially expressed RNAs were considered as candidate genes to construct the multigene risk score (MGRS).

### Development and assessment of MGRS

Penalized regression methods, including least absolute shrinkage and selection operator (LASSO), ALASSO (adaptive LASSO), and elastic net (EN), were used to screen the variables (mRNAs and lncRNAs) to construct the MGRS. The tuning parameter λ of the penalized regression methods was determined by the rule of minimum mean cross-validated error. We performed the stepwise variables selection strategy in the Cox model to remove genes that were not significant predictors in the absence of the constraint imposed by the penalty [72]. To reduce the sensitivity of the variable selection procedure to the cross-validation process in the penalized regression, we repeated the selection strategy (penalized Cox regression+stepwise) 100 times with different cross-validation folds [72]. We used the set of 100 times penalized Cox regression+stepwise selected genes to construct a multivariable Cox model and defined its prognostic index as the MGRS. Patients from the different dataset were divided into MGRS-high risk and MGRS-low risk groups according to whether their MGRS was above or below the cutoff point, which was defined as the mean of the MGRSs in the training dataset.

To evaluate the independent role of the MGRS in prognosis, data stratification analysis and multivariable Cox regression analysis were performed. For the stratification analysis, Kaplan–Meier and log-rank tests were applied to compare the difference in survival between the MGRS-high risk and MGRS-low risk groups in each stratum. Then, we constructed a nomogram based on the results of the multivariable Cox analysis for clinical use [73]. The performance of the nomogram that integrated the IPI and MGRS in predicting 2-year survival was evaluated by its discrimination, calibration, and clinical usefulness [32]. Discrimination was measured by ROC curves [32, 74] at 2 years after diagnosis. Model calibration was assessed by comparing the observed 2-year survival rate with the mean of the predicted 2-year survival rate [32]. The observed 2-year survival rate was estimated using the Kaplan-Meier method. A model was considered well calibrated if the predicted 2-year survival rate was close to the observed one. The clinical usefulness of the model was assessed by decision curve analysis [32]. We also assessed the added value from the MGRS in the 2-year survival prediction by comparing the performance of the models with and without MGRS. The category-free net reclassification index (NRI>0) was used to quantify the ability of the IPI+MGRS model to correctly reclassify patients (survival or death at 2-year) comparing with that of the IPI model [32]. The NRI (>0) ranged from −2 to +2. A positive value of NRI (>0) indicated improved reclassification ability of the IPI+MGRS model.

To further investigate the generalizability of the MGRS, we also performed sensitivity analysis on three subsets of the validation dataset: ComBatData1, which contained the biggest dataset GSE31312; ComBatData2, which contained validation dataset without GSE31312; and ComBatData3, which contained the validation dataset without the datasets with sample size less than 100 (GSE98588 and GSE23501). The added value offered by MGRS was assessed in the sensitivity analysis.

### Functional enrichment analysis

To explore the functional role of MGRS in patients with DLBCL, we constructed a co-expression network by weighted correlation network analysis [75]. Then, the correlation between the MGRS and each module in the co-expression network was estimated to identify highly-correlated modules. The genes in the highly-correlated module were functionally annotated by GO and KEGG pathway enrichment analysis [76]. Pathways with adjusted p <0.05 and nominal p <0.01 were considered statistically significant.

All the statistical analyses were performed using R-3.5.1 and SAS (version 9.4). A flow chart of the statistical process is given in Figure 1.

## Supplementary Material

Supplementary Table 1

Supplementary Figure 1
