# Supplementary material for: Transcriptome profiling reveals an integrated mRNA–lncRNA signature with predictive value for long-term survival in diffuse large B-cell lymphoma
Source: Aging (Albany NY). 2020 Nov 18;12(22):23275–95. doi: 10.18632/aging.104100 (PMC7746345; doi:10.18632/aging.104100)
Supplement: Supplementary Table 1 [file aging-12-104100-s001..pdf]

SUPPLEMENTARY TABLES

Supplementary Table 1. Baseline characteristics of the training and validation datasets.

Variables	Training dataset	Validation dataset	Components of validation dataset			
	GSE10846 (n=412)	ComBatData* (n=832)	GSE31312 (n=470)	GSE23501 (n=69)	GSE87371 (n=221)	GSE98588 (n=72)
**age,year**						
≤60	188 (45.6%)	369(44.4%)	200 (42.6%)	28 (40.6%)	115 (52.0%)	26 (36.1%)
>60	224 (54.4%)	463 (55.6%)	270 (57.4%)	41 (59.4%)	106 (48.0%)	46 (63.9%)
**Sex**						
Female	172 (41.7%)	323 (38.8%)	199 (42.3%)	19 (27.5%)	105 (47.5%)	-
Male	222 (53.9%)	437 (52.5%)	271 (57.7%)	50 (72.5%)	116 (52.5%)	-
Unknown	18 (4.4%)	72 (8.7%)	0 (0.0%)	0 (0.0%)	0 (0.0%)	72 (100%)
**Stage**						
I/II	188 (45.6%)	314 (37.8%)	220 (46.8%)	-	71 (32.1%)	23 (31.9%)
III/IV	217 (52.7%)	427 (51.3%)	228 (48.5%)	-	150 (67.9%)	49 (68.1%)
Unknown	7 (1.7%)	91 (10.9%)	22 (4.7%)	69 (100%)	0 (0.0%)	0 (0.0%)
**No.of extranodal sites**						
<2	351 (85.2%)	421 (50.6%)	366 (77.9%)	-	-	55 (76.4%)
≥2	30 (7.3%)	121 (14.5%)	104 (22.1%)	-	-	17 (23.6%)
Unknown	31 (7.5%)	290 (34.9%)	0 (0.0%)	69 (100%)	221 (100%)	0 (0.0%)
**LDH**						
0	173 (42.0%)	179 (21.5%)	148 (31.5%)	-	-	31 (43.1%)
1	177 (43.0%)	319 (38.3%)	278 (59.1%)	-	-	41 (56.9%)
Unknown	62 (15.0%)	334 (40.2%)	44 (9.4%)	69 (100%)	221 (100%)	0 (0.0%)
**ECOG**						
<2	295 (71.6%)	432 (51.9%)	374 (79.6%)	-	-	58 (80.6%)
≥2	93 (22.6%)	110 (13.2%)	96 (20.4%)	-	-	14 (19.4%)
Unknown	24 (5.8%)	290 (34.9%)	0 (0.0%)	69 (100%)	221 (100%)	0 (0.0%)
**IPI**						
0-2	228 (55.4%)	464(55.8%)	274 (58.3%)	33 (47.8%)	119 (53.8%)	38 (52.8%)
3-5	92 (22.3%)	318 (38.2%)	150 (31.9%)	32 (46.4%)	102 (46.2%)	34 (47.2%)
Unknown	92 (22.3%)	50 (6.0%)	46 (9.8%)	4 (5.8%)	0 (0.0%)	0 (0.0%)
**Subtype**						
GCB	182 (44.2%)	372 (44.7%)	248 (52.8%)	40 (58.0%)	84 (38.0%)	-
Non-GCB	230 (55.8%)	388 (46.6%)	222 (47.2%)	29 (42.0%)	137 (62.0%)	-
Unknown	0(0.0%)	72 (8.7%)	0 (0.0%)	0 (0.0%)	0 (0.0%)	72 (100%)
**Overall survival,year**						
median survival time	6.94	7.61	6.86	-	-	-

*ComBatData is an integrated dataset, and it combined GSE31312, GSE23501,GSE87371 and GSE98588 using ComBat method.

Abbreviation: Stage, Ann Arbor stage; LDH, lactate dehydrogenase; ECOG, Eastern Cooperative Oncology Group; IPI, International Prognostic Index; GCB, germinal center B-cell-like.
