# Supplementary figures and images for: Transcriptome profiling reveals an integrated mRNA–lncRNA signature with predictive value for long-term survival in diffuse large B-cell lymphoma
Source: Aging (Albany NY). 2020 Nov 18;12(22):23275–95. doi: 10.18632/aging.104100 (PMC7746345; doi:10.18632/aging.104100)

## SUPPLEMENTARY FIGURE

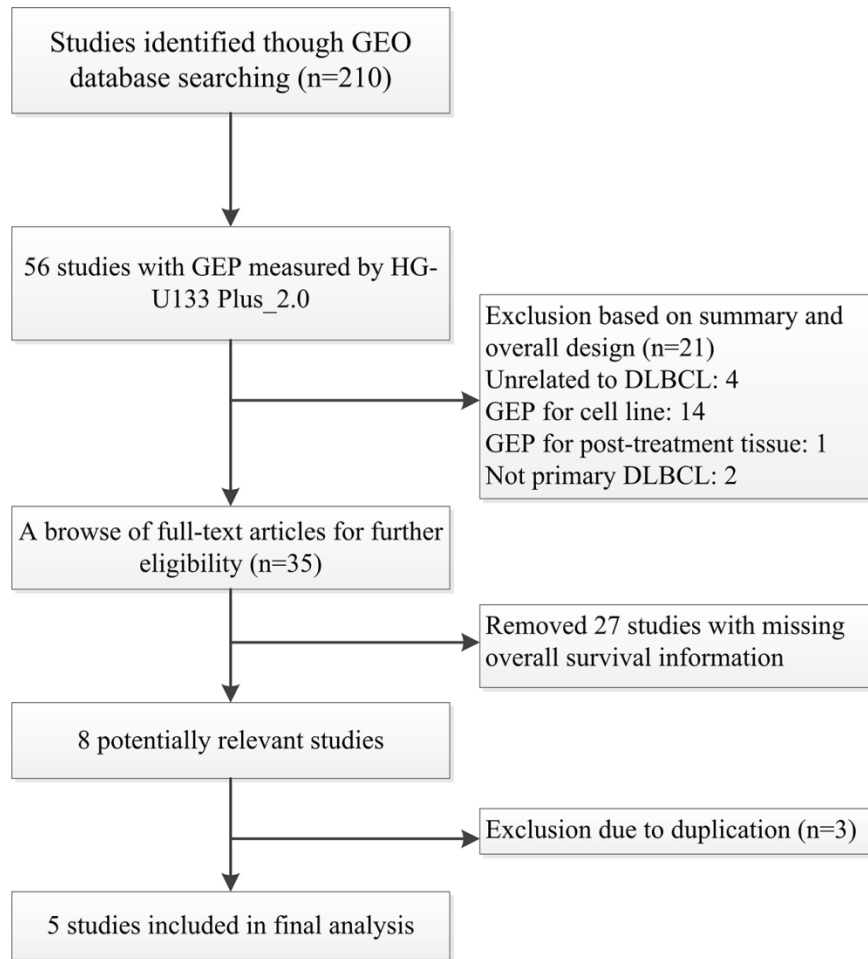

**Supplementary Figure 1. Flow chart of the study selection process.**

Supplement: Supplementary Figure 1 [file aging-12-104100-s002..pdf]
